# Second sound in the crossover from the Bose-Einstein condensate to the Bardeen-Cooper-Schrieffer superfluid

**DOI:** 10.1038/s41467-021-27149-z

**Published:** 2021-12-06

**Authors:** Daniel K. Hoffmann, Vijay Pal Singh, Thomas Paintner, Manuel Jäger, Wolfgang Limmer, Ludwig Mathey, Johannes Hecker Denschlag

**Affiliations:** 1 Institut für Quantenmaterie and Center for Integrated Quantum Science and Technology (IQST), Universität Ulm, D-89069 Ulm, Germany; 2 Institut für Theoretische Physik, Leibniz Universität Hannover, Appelstraße 2, 30167 Hannover, Germany; 3 Institut für Laserphysik, Zentrum für Optische Quantentechnologien, Universität Hamburg, 22761 Hamburg, Germany; 4 The Hamburg center for Ultrafast Imaging, Universität Hamburg, Luruper Chaussee 149, 22761 Hamburg, Germany

**Keywords:** Phase transitions and critical phenomena, Quantum fluids and solids, Bose-Einstein condensates

## Abstract

Second sound is an entropy wave which propagates in the superfluid component of a quantum liquid. Because it is an entropy wave, it probes the thermodynamic properties of the quantum liquid. Here, we study second sound propagation for a large range of interaction strengths within the crossover between a Bose-Einstein condensate (BEC) and the Bardeen-Cooper-Schrieffer (BCS) superfluid, extending previous work at unitarity. In particular, we investigate the strongly-interacting regime where currently theoretical predictions only exist in terms of an interpolation in the crossover. Working with a quantum gas of ultracold fermionic ^6^Li atoms with tunable interactions, we show that the second sound speed varies only slightly in the crossover regime. By varying the excitation procedure, we gain deeper insight on sound propagation. We compare our measurement results with classical-field simulations, which help with the interpretation of our experiments.

## Abstract (web summary)

The second sound is an entropy oscillation propagating with constant pressure. Here the authors demonstrate the variation of the second sound wave in the crossover from the Bose-Einstein condensate to the Bardeen-Cooper-Schrieffer superfluid using 6Li.

## Introduction

Second sound is a transport phenomenon of quantum liquids that emerges below the critical temperature for superfluidity *T*_c_^1–3^. It was experimentally discovered^4^ in 1944 in He II^5^ and was described with a hydrodynamic two-fluid model^2,6–8^ which treats He II as a mixture of a superfluid (SF) and a normal fluid (NF). The SF component has no entropy and flows without dissipation. The NF component carries all the entropy and has non-zero viscosity. In the limit of vanishing temperature *T* → 0, the two-fluid model predicts that first sound (i.e., standard sound waves) corresponds to a propagating pressure oscillation with constant entropy, while second sound is an entropy oscillation propagating at constant pressure^8^.

The properties of a SF naturally depend on parameters such as its temperature and the interaction strength between its particles. With the advent of ultracold quantum gases, with tunable interactions, these dependencies can now be studied. In particular, an ultracold fermionic quantum gas with a tunable Feshbach resonance offers a unique opportunity to access various sorts of superfluidity in one system, ranging continuously between a Bose-Einstein condensate (BEC) of bosonic molecules, a resonant SF, and a SF gas of Cooper pairs (BCS superfluid)^9–11^. In the experiment, this is done by tuning the interaction parameter ${({k}_{{{{{{{{\rm{F}}}}}}}}}a)}^{-1}$, where *a* is the scattering length, ${k}_{{{{{{{{\rm{F}}}}}}}}}=\sqrt{2m{E}_{{{{{{{{\rm{F}}}}}}}}}}/\hslash$ the Fermi wavenumber, *E*_F_ is the Fermi energy, and *m* the atomic mass.

A large range of thermodynamical properties of the BEC-BCS crossover has been studied, e.g., in refs. ^11–19^. This includes experiments on first sound (see e.g., ref. ^16^) and second sound. Second sound has recently been observed by Sidorenkov et al.^20^ in a unitary Fermi gas and by Ville et al.^21^ and Christodoulou et al.^22^ in a two-dimensional bosonic SF. Second sound was also possibly present in an experiment by Meppelink et al.^23^ as pointed out in ref. ^3^.

Here, we experimentally investigate how second sound changes across the BEC–BCS crossover. This is important, because full theoretical calculations are not yet available for the entire strongly interacting regime. Nevertheless, comparing our measurements to existing calculations and interpolations we find reasonable agreement. In particular, c-field simulations in the BEC regime match quite well the observed wave dynamics in experiments at interaction strengths of up to ${({k}_{{{{{{{{\rm{F}}}}}}}}}a)}^{-1}=1$. In addition, we investigate experimentally and theoretically the system response when modifying the excitation scheme. As second sound is mainly an entropy wave and first sound is mainly a pressure wave, different excitation schemes give rise to different responses for first and second sound. This helps for separating the generally weak second sound signals from the first sound ones. We find that this separation works especially well when first sound is excited as a density dip wave packet. For this case, we were able to quantitatively compare the amplitudes of first and second sound and compare the results with a prediction.

## Results

### Experimental details

Our experiments are carried out with a balanced, two-component ultracold gas of fermionic ^6^Li atoms in the two lowest hyperfine states $\left|F,{m}_{F}\right\rangle =\left|1/2,\pm \!1/2\right\rangle$ of the electronic ground state. The gas is confined by a combined magnetic and optical dipole trap with a trap depth of *U*_0_ ≈ 1 μK × *k*_B_, for details see ref. ^24,25^. The trap is nearly harmonic and cylindrically symmetric with trapping frequencies *ω*_*r*_ = 2*π* × 305 Hz and *ω*_*x*_ = 2*π* × 21 Hz. The temperature and the particle density are controlled by evaporative cooling. In the experiments the temperature ranges approximately from 0.13 *T*_F_ to 0.30 *T*_F_, where ${T}_{{{{{{{{\rm{F}}}}}}}}}={E}_{{{{{{{{\rm{F}}}}}}}}}/{k}_{{{{{{{{\rm{B}}}}}}}}}=\hslash \bar{\omega }{(3N)}^{1/3}/{k}_{{{{{{{{\rm{B}}}}}}}}}$ is the Fermi temperature, $\bar{\omega }={\left({\omega }_{x}{\omega }_{r}^{2}\right)}^{1/3}$ is the geometric mean of the trapping frequencies and *N* is the total number of atoms. The scattering length *a* is tunable with an external magnetic field *B* via a magnetic Feshbach resonance at 832 G^26^.

**Fig. 1:**
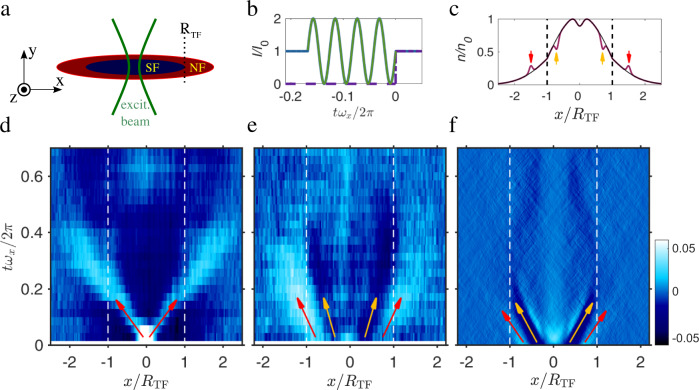
Sound excitation in a trapped superfluid Fermi gas in the vicinity of the BEC-BCS crossover. **a** Set-up: A focussed, intensity-modulated, blue-detuned laser beam excites sound waves in the cigar-shaped atom cloud. **b** Two different modulation sequences of the laser intensity. Purple dashed line: step excitation. Green solid line: heat pulse. The time *t* is given in units of the axial trapping period 2*π*/*ω*_*x*_. **c** Sketch of a bimodal density distribution of a trapped BEC (purple line) at *y* = *z* = 0. At the center of the trap a blue detuned beam produces a dimple in the potential. Modulating the beam intensity produces first sound waves (red arrows) and second sound (orange arrows) waves. Second sound reduces the local density of the cloud, while for first sound a density peak emerges. The thin black line shows the profile of the unperturbed cloud. Please note that the crests and troughs of the waves are shown in an exaggerated way for better visibility. **d** The false color plot shows the measured local change in the density ${{\Delta }}\bar{n}(x,t)$ as a function of axial position *x* and time *t*. Here, ${({k}_{{{{{{{{\rm{F}}}}}}}}}a)}^{-1}=(1.61\pm 0.05)$ at *B* = 735 G and *T*/*T*_c_ = (0.80 ± 0.15). After excitation, two wave packets (bright traces, marked with red arrows) propagate with first sound velocity *u*_1_ towards the edges of the cloud. The excitation method predominantly excites first sound. Second sound is present as well but is barely discernible here. **e** Propagation of first sound waves (bright traces, marked with red arrow) and second sound waves (dark traces, marked with orange arrows) after excitation with sinusoidal pulse of (**b**). All other settings are the same as in (**d**). **f** Simulated sound propagation for the same parameters as in (**e**). The orange arrows mark the propagating second sound and the red arrows the first sound, respectively.

To excite sound modes in the system, we focus a blue-detuned 532 nm laser onto the trap center following the approaches introduced in refs. ^16,20,27^ (Fig. 1a). The laser beam is aligned perpendicularly to the optical dipole trap and produces a repulsive potential barrier of *U*_ex_ ≈ 0.2 *U*_0_. At its focus, the beam has a waist of about 20 μm, which is comparable to the cloud size in the radial direction. To excite sound waves, the height of this additional potential is modulated. The excited sound modes generally exhibit contributions from both first and second sound^28–30^. However, it is possible to generate preferentially either one of the two sound modes by adapting the excitation method.

To excite primarily first sound, we abruptly switch on the excitation laser beam (Fig. 1b), similarly as for the first experiments on sound propagation in a dilute BEC^27^. This applies pressure on the cold cloud on both sides of the laser beam and creates two density wave packets (Fig. 1c) which propagate out in opposite directions along the axial trap axis with the speed *u*_1_. In the experiments, we detect these waves with the help of absorption imaging by measuring the density distribution of the atomic cloud as a function of time.

Figure 1d shows such density waves for an experiment at ${({k}_{{{{{{{{\rm{F}}}}}}}}}a)}^{-1}\approx (1.61\pm 0.05)$, *B* = 735 G and a temperature of *T* = (220 ± 30) nK = (0.30 ± 0.06) *T*_F_, which corresponds to *T* = (0.80 ± 0.15) *T*_c_, where *T*_c_ is the critical temperature. For the given interaction strength, we used *T*_c_ = 0.37 *T*_F_ (see Supplementary Note 1).

Figure 1d is a time-ordered stack of one-dimensional line density profiles of the atom cloud^31^. Each profile was obtained by integrating the measured column density of the atomic cloud along the transverse (i.e., *y*-) direction (see “Methods” for details). The time-ordered stack shows the propagation of the sound waves along the axial direction *x* as a function of time. We observe two density wave packets which propagate with first sound velocity from the trap center towards the edge of the cloud (two bright traces, marked with red arrows). The propagating waves produce a density modulation of only a few percent of the peak density and can be considered as a weak perturbation of the system. To obtain the speed of sound, we examine how the center position of each wave packet changes with time. The center positions are determined via a Gaussian fit. Please note that the central sound speed is determined over a local area of the cloud where the density varies by about 30%. As a consequence, the measured sound speed should be considered a mean value. From Fig. 1d we obtain *u*_1_ = (17.2 ± 3) mm/s near the trap center. Our analysis shows that the sound propagation slows down as the pulse approaches the edge of the cloud where the particle density decreases. In the following, we focus on the sound speed close to the trap center.

To primarily excite second sound, we sinusoidally modulate the intensity of the excitation beam for 7 ms with a modulation frequency of *ω*_ex_ = 2*π* × 570 Hz ≈ 2*ω*_*r*_ and a modulation amplitude of Δ*U* ≈ 0.2 *U*_0_. This parametrically heats the gas in radial direction (Fig. 1b). Subsequent thermalization via collisions occurs within a few milliseconds. As a consequence a local depletion of the SF density is created, which is filled with normal gas, forming a region of increased entropy (Fig. 1c). This gives rise to two wave packets which propagate outwards along the axial direction with the speed of second sound. Figure 1e shows corresponding experimental data where we measure the local density distribution as in Fig. 1d. The second sound wave appears here as a density dip (dark traces, marked with orange arrows). A clear indication that the dark trace corresponds to second sound is the fact that it vanishes at the Thomas-Fermi radius *R*_TF_ ≈ 110 μm where the SF fraction vanishes. Second sound only propagates inside the SF phase.

Besides a second sound wave, the excitation also produces a first sound wave (bright traces, marked with red arrows) which propagates faster than the second sound wave and travels beyond the Thomas-Fermi radius. The first sound wave is broader than in Fig. 1d, which can be mainly explained by the longer excitation pulse. To obtain *u*_2_ we measure the time-dependent position of the minimum of each dark trace, which is again determined via a Gaussian fit. For Fig. 1e we obtain *u*_2_ = (5.1 ± 1.1) mm/s. We note that for the small excitation amplitudes in our experiments (see colorbar in Fig. 1d–e), we do not observe the asymmetric sound wave distortions reported in ref. ^23^. These distortions were also absent in ref. ^20^.

Figure 1f shows numerical simulations of our experiment applying a dynamical c-field method^32^ (see Supplementary Note 2 for detailed information on the method). The dimer scattering length^33^ is *a*_dd_ = 0.6*a* and we assume all fermionic atoms to be paired up in molecules. To compare the simulations with the experimental results we choose the same values of ${({k}_{{{{{{{{\rm{F}}}}}}}}}a)}^{-1}$ and the same central density of the trapped gas as in the experiment. The theory value for *u*_2_ is (5.7 ± 0.05) mm/s in agreement with the experimental value (5.1 ± 1.1) mm/s.

### Interaction strength dependence of second sound

**Fig. 2:**
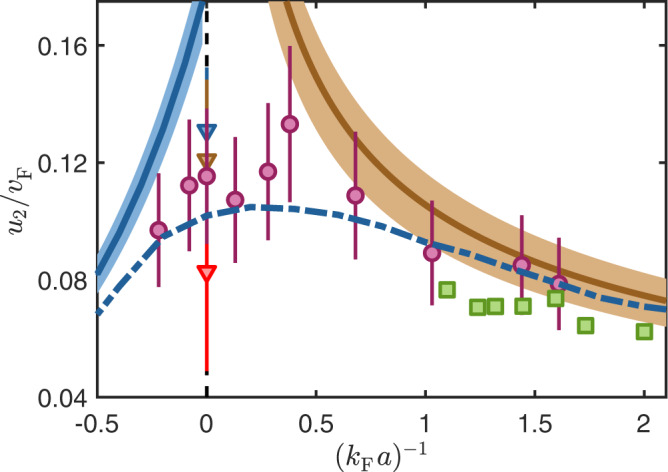
Second sound velocity *u*_2_ as a function of interaction strength. The purple circles depict measured data for temperatures in the range *T* = 105−230 nK which corresponds to *T*/*T*_c_ = 0.66−0.84 (see Supplementary Note 1). The error bars are due to statistical uncertainties. The brown and blue solid line show hydrodynamic predictions for the BEC and BCS regime at *T* = 0.75*T*_c_, respectively (see Supplementary Note 3). The shaded areas mark the second sound velocity in the temperature range of the experiments. The blue dash-dotted line shows a theoretical prediction of second sound in the crossover^28^ for a homogeneous gas at *T*/*T*_c_ = 0.75. It interpolates between the results from hydrodynamic theory in the BEC and BCS regime. The green squares are results of our numerical c-field simulations which are consistent with both, analytic and experimental results. For comparison we also show the second sound velocity on the resonance measured in ref. ^20^ at the temperatures *T*/*T*_c_ = 0.65 (blue triangle), *T*/*T*_c_ = 0.75 (brown triangle), and *T*/*T*_c_ = 0.85 (red triangle).

We now perform measurements of second sound speed in the region $(-0.22\pm 0.04) \, < \,{({k}_{{{{{{{{\rm{F}}}}}}}}}a)}^{-1} \, < \,(1.61\pm 0.05)$ of the BCS-BEC crossover. These are shown in Fig. 2 along with theoretical predictions. The second sound velocity *u*_2_ is given in units of the Fermi velocity ${v}_{{{{{{{{\rm{F}}}}}}}}}=\hslash {k}_{{{{{{{{\rm{F}}}}}}}}}^{{{\rm{hom}}}}/m$. Here, the Fermi wavenumber ${k}_{{{{{{{{\rm{F}}}}}}}}}^{{{\rm{hom}}}}= {\left(3{\pi }^{2}{n}_{0}\right)}^{1/3}$ is determined from the 3D peak density close to the trap center. The peak density is deduced using the inverse Abel transformation^34^ and a self-consistent mean field calculation (see Supplementary Note 4). We have verified that in the BEC regime our mean field calculation gives similar results for the peak density when we input the trapping frequencies, the temperature, the scattering length and the total number of particles. The blue dash-dotted line is a calculation from ref. ^28^, based on a hydrodynamic description in a homogeneous gas for the limiting cases of the BEC and the BCS regime and unitarity. To connect these regimes, the results are interpolated across the crossover, bridging the range $|{({k}_{{{{{{{{\rm{F}}}}}}}}}a)}^{-1}| \, < \, 1$. The blue solid and the brown solid lines are our analytic hydrodynamic calculations which are valid in the BCS and BEC limit, respectively (see Supplementary Note 3). For comparison, we show the results of the numerical c-field simulations (green squares), which agree with both, analytic description and experimental results. Despite the large error bars the measurements indicate an increase of *u*_2_ when approaching unitarity from the BEC side, in agreement with the theoretical results.

In general, second sound can only propagate in the SF phase of the gas. It is therefore natural to ask how the SF density *n*_s_ and the speed of second sound *u*_2_ are related. Using our measurements for *u*_2_ we can roughly determine the relationship between *n*_s_ and *u*_2_ for our temperature *T* and ${({k}_{{{{{{{{\rm{F}}}}}}}}}a)}^{-1} \, > \, 1.5$, since in this regime the SF density can be estimated. For this, we carry out self-consistent mean field calculations to determine the density distributions of the SF and the NF for an interacting BEC in the trap (see Supplementary Note 4). As an important input into these calculations we make use of the Thomas-Fermi radius which we have measured in the second sound experiments (the measured Thomas-Fermi radii can be found in Supplementary Note 1). As an example, from the measurement at ${({k}_{{{{{{{{\rm{F}}}}}}}}}a)}^{-1}=(1.61\pm 0.05)$ we determine the peak SF fraction to be *n*_s0_/*n*_0_ = 0.98 close to the trap center at maximum density (see Supplementary Note 4), where the local ${({k}_{{{{{{{{\rm{F}}}}}}}}}^{{{\rm{hom}}}}a)}^{-1}=(1.06\pm 0.05)$ and $T/{T}_{{{{{{{{\rm{c}}}}}}}}}^{{{\rm{hom}}}}=(0.40\pm 0.15)$, with ${T}_{{{{{{{{\rm{c}}}}}}}}}^{{{\rm{hom}}}}= 0.21{T}_{{{{{{{{\rm{F}}}}}}}}}^{{{\rm{hom}}}}$ and ${T}_{{{{{{{{\rm{F}}}}}}}}}^{{{\rm{hom}}}}={\hslash }^{2}{({k}_{{{{{{{{\rm{F}}}}}}}}}^{{{\rm{hom}}}})}^{2}/2m{k}_{{{{{{{{\rm{B}}}}}}}}}$. For comparison, for a homogeneous weakly interacting BEC with a SF fraction close to unity the temperature would need to be $T\ll {T}_{{{{{{{{\rm{c}}}}}}}}}^{{{\rm{hom}}}}$, according to ${n}_{{{{{{{{\rm{s}}}}}}}}}/n=1-{(T/{T}_{{{{{{{{\rm{c}}}}}}}}}^{{{\rm{hom}}}})}^{3/2}$. At unitarity, by contrast, the SF fraction reaches unity already at $T/{T}_{{{{{{{{\rm{c}}}}}}}}}^{{{\rm{hom}}}}\approx 0.55$, as shown by Sidorenkov et al.^20^. As expected, this comparison shows that for a given $T/{T}_{{{{{{{{\rm{c}}}}}}}}}^{{{\rm{hom}}}}$ the SF fraction grows with interaction strength.

### Tuning the sound mode excitation

In the following we investigate how the SF gas responds to different excitation protocols^28–30^. For this, we vary the excitation scheme, the excitation frequency and amplitude. By observing the corresponding response of the system we gain additional insights into the nature of first and second sound.

**Fig. 3:**
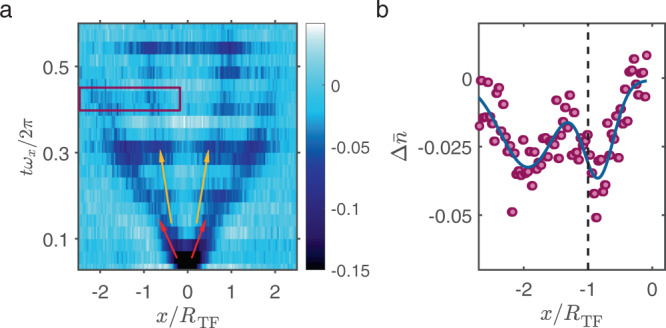
Comparing signal strength of first and second sound. **a** Sound excitation experiment at ${({k}_{{{{{{{{\rm{F}}}}}}}}}a)}^{-1}=(1.61\pm 0.05)$ and at a temperature of *T*/*T*_c_ = (0.80 ± 0.15). In contrast to Fig. 1d, first sound (red arrows) and second sound (orange arrows) are now visible simultaneously. For *tω*_*x*_/2*π* < 0.15 first and second sound waves overlap and therefore cannot be distinguished from each other. **b** shows ${{\Delta }}\bar{n}$ for *tω*_*x*_/2*π* = 0.43. We fit the center position of each of the two sound waves using a Gaussian function (solid line).

In Fig. 3a we show the evolution of the system after a step pulse excitation at *B* = 735 G and Δ*U* = 0.3*U*_0_, in which both, first and second sound are excited. In contrast to the experiment in Fig. 1d, the laser beam is abruptly switched off - not on. As a consequence, the wave packets of both first and second sound now correspond to dips in the particle density. In Fig. 3b we show the density distribution for the time and position range indicated by the purple rectangle in Fig. 3a. From a fit of two Gaussian dips to the two wave packets, we determine an amplitude ratio of *W*_2_/*W*_1_ ≈ 1.1. This result approximately matches the predictions of refs. ^29,30^ (see also Supplementary Note 3), where the response of both, a weakly and a strongly interacting molecular Bose gas has been studied. For an interaction parameter of ${({k}_{{{{{{{{\rm{F}}}}}}}}}a)}^{-1}=2$, the prediction yields *W*_2_/*W*_1_ = 0.9, which is of similar magnitude as our result.

**Fig. 4:**
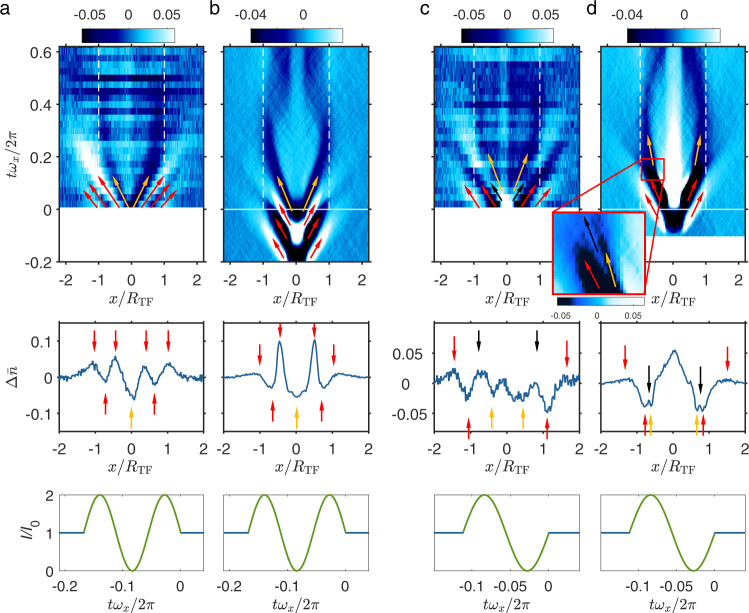
Sound excitation with different modulation sequences. **a**, **c** ${{\Delta }}\bar{n}(x,t)$ data at *ω*_ex_ = 0.61*ω*_*r*_, Δ*U* = 0.3*U*_0_ and ${\left({k}_{{{{{{{{\rm{F}}}}}}}}}a\right)}^{-1}=(1.61\pm 0.05)$ for a modulation of 1.5 cycles and of 1 cycle, respectively. The excitation pulse excites both, first and second sound waves. **b**, **d** ${{\Delta }}\bar{n}(x,t)$ from numerical c-field simulations. Top row: False color images of ${{\Delta }}\bar{n}(x,t)$. First and second sound waves are marked with red and orange arrows, respectively. The inset in (**d**) is an enlargement, showing how the calculated bright first sound wave (black arrow) is damped when it crosses the second sound wave (orange arrow). Mid row: Shown is ${{\Delta }}\bar{n}$ for *tω*_*x*_/2*π* = 0 (**a**, **b**) and for *tω*_*x*_/2*π* = 0.15 (**c**, **d**). Bottom row: Applied excitation scheme.

Next, we study the system response when modifying the excitation scheme. For this, we vary the excitation frequency and the number of modulation cycles. Figure 4a–d shows the system response for a modulation frequency of *ω*_ex_ = 0.61*ω*_*r*_, so that parametric heating is rather suppressed and coupling to first sound is enhanced as compared to the measurement shown in Fig. 1e. While Fig. 4a, b corresponds to a 1.5 cycle modulation, Fig. 4c, d corresponds to a 1 cycle modulation.

The numerical simulations in Fig. 4b, d demonstrate how the excitation pattern produces a corresponding wave train of first sound. Once the simulated waves of first sound have propagated beyond the Thomas-Fermi radius, they diffuse out and lose signal strength. In the simulation, the diffusion of the first sound wave train is stronger than in the experiment. This might be explained by the choice of the discretization length which was used in the simulations (see Supplementary Note 2B). The first sound wave train is always followed by a single dark second sound wave packet.

The experimental data in Fig. 4a agree reasonably well with the simulation in Fig. 4b. Comparing Fig. 4c and d, there seems to be a discrepancy. In the experiment clearly a first sound wave train, consisting of two bright traces and one dark trace, propagates beyond the Thomas-Fermi radius. In the simulation, however, the second bright trace of the wave train, which is clearly visible in the center, disappears before it reaches the Thomas-Fermi radius (black arrow). The corresponding damping happens when the bright sound trace crosses the dark second sound trace (see also insert in Fig. 4d). The reason for the damping is that first sound waves diffuse in a thermal environment. This diffusion, however, is apparently too strong in the simulation results, which is probably a consequence of constraints in the numerical resolution (for more details see Supplementary Note 2B).

In conclusion, we have studied second sound propagation in an ultracold Fermi gas of ^6^Li atoms throughout the BEC-BCS crossover for a finite temperature of *T* ≈ 0.75 *T*_c_. We find the second sound velocity to vary only slightly across the BCS-BEC crossover, which is in agreement with an interpolation of hydrodynamic theory^28^. In the BEC regime, the results match numerical predictions based on c-field simulations.

In addition, we investigate the response of the SF gas on various excitation pulse shapes, ranging from gentle local heating to an abrupt kick. While a sinusoidal excitation pulse leads to a corresponding wave train for the first sound, it only produces a single pulse for the second sound. In the future, it will be useful to extend our measurements in the strongly interacting regime to a larger range of temperatures below *T*_c_. Since the second sound velocity is related to the local SF density, these measurements can help to determine the correlation between the SF density and the temperature in the strongly interacting regime.

## Methods

### Calculating ${{\Delta }}\bar{n}$ from the density profiles

Each of the experimental sound propagation images in Figs. 1d–e, 3a, 4a and c is a time-ordered stack of one-dimensional line density profiles ${{\Delta }}\bar{n}(x,t)$ of the atom cloud. A one-dimensional line density profile *n*(*x*, *t*) is produced as follows: After sound excitation has ended and after an additional propagation time *t*, we take an absorption image of the atom cloud to obtain the column density distribution *n*_ex_(*x*, *y*, *t*). Next, we integrate the absorption image along the *y-*axis (which is perpendicular to the symmetry axis of the cigar-shaped atom cloud) to obtain a one-dimensional line density profile *n*_ex_(*x*, *t*). To reduce noise, we average 15 density profiles and obtain ${\bar{n}}_{{{{{{{{\rm{ex}}}}}}}}}(x,t)$. We repeat this procedure for an unperturbed cloud to obtain $\bar{n}(x)$. By subtracting the two density profiles from each other we obtain ${{\Delta }}\bar{n}(x,t)=({\bar{n}}_{{{{{{{{\rm{ex}}}}}}}}}(x,t)-\bar{n}(x))/\bar{n}(0)$.

## Supplementary information

Supplementary Information

## Data Availability

The data for the measured temperatures and Thomas-Fermi radii are provided in Supplementary Note 1.

## Code availability

The simulation codes and simulated data are available at https://zenodo.org/record/5572570^35^.
