# Supplementary material for: Second sound in the crossover from the Bose-Einstein condensate to the Bardeen-Cooper-Schrieffer superfluid
Source: Nat Commun. 2021 Dec 6;12:7074. doi: 10.1038/s41467-021-27149-z (PMC8648831; doi:10.1038/s41467-021-27149-z)
Supplement: Supplementary file 1 — Supplementary Information [file 41467_2021_27149_MOESM1_ESM.pdf]

SUPPLEMENTARY INFORMATION

Supplementary Note 1: Temperatures to the measurements in Fig. 1 and Fig. 2

In this section we present the temperatures to the measurements shown in Fig. 1 and Fig. 2 (see Supp. Table 1). We determine the temperatures by fitting a second order virial expansion of the density distribution at the wings of the cloud[1]. To compare the absolute temperature with T_{c} for various interaction strengths we use values for T_{c} as shown in Supp. Figure 1.

T_{c} is not precisely known yet in the strongly interacting regime. In the limit of the BEC regime the BEC mean-field model should give accurate values for critical temperature. Closer towards the resonance we expect the diagrammatic t-matrix calculation to provide quite good values[2]. For the range in between ($0.5 < (k_{\mathrm{F}}a)^{-1} < 3$) we linearly interpolate between both T_{c} curves.

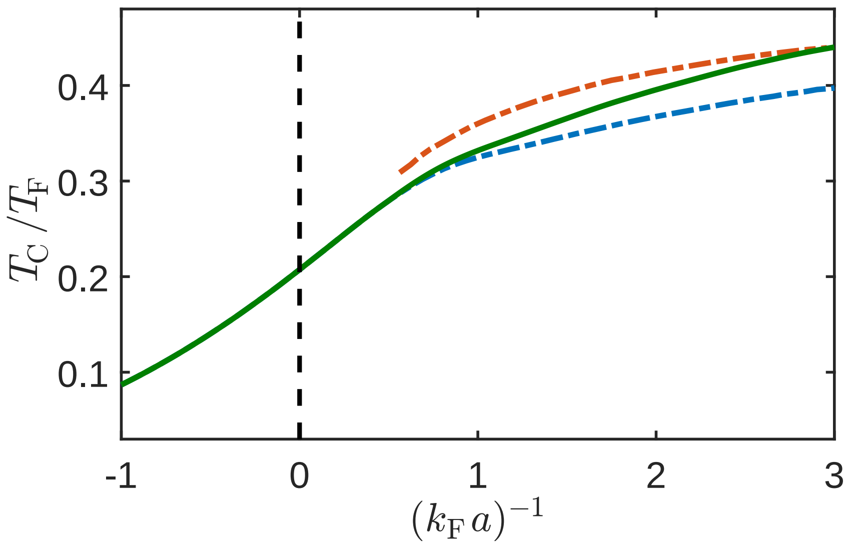

Supplementary Figure 1. **Critical temperature T_{c} in units of T_{F} as a function of $(k_{\mathrm{F}}a)^{-1}$ for a harmonically trapped Fermi gas.** The blue dash-dotted line shows a diagrammatic t-matrix calculation and the orange dash-dotted line a calculation based on a BEC mean-field model[2]. The green straight line interpolates linearly between the two approaches. In the BEC limit of noninteracting molecules T_{c} is given by $T_{\mathrm{c}} = 0.94\hbar(\omega_x\omega_r^2 N)^{1/3}$ and therefore $T_{\mathrm{c}}/T_{\mathrm{F}} = 0.517$.

For the measurements on the resonance we compared our result with the temperature determined using the equation of state from Ref.[3]. We find reasonable agreement between

the temperatures obtained from the two approaches with deviations on the order of $5-10\%$.

$(k_\mathrm{F}a)^{-1}$	T [nK]	T/T_F	$T_\mathrm{c}/T_\mathrm{F}$	T/T_c	R_TF [μm]
-0.22 ± 0.04	105 ± 23	0.13 ± 0.04	0.176	0.74 ± 0.24	96 ± 5
-0.08 ± 0.03	106 ± 22	0.13 ± 0.04	0.196	0.66 ± 0.21	107 ± 5
0 ± 0.02	158 ± 28	0.17 ± 0.05	0.207	0.82 ± 0.24	108 ± 5
0.13 ± 0.03	140 ± 30	0.18 ± 0.05	0.226	0.80 ± 0.22	121 ± 5
0.28 ± 0.04	140 ± 30	0.19 ± 0.05	0.249	0.76 ± 0.20	156 ± 5
0.38 ± 0.04	190 ± 30	0.22 ± 0.06	0.263	0.84 ± 0.22	153 ± 5
0.68 ± 0.05	190 ± 30	0.24 ± 0.06	0.302	0.79 ± 0.19	139 ± 5
1.03 ± 0.05	200 ± 30	0.25 ± 0.06	0.334	0.75 ± 0.17	124 ± 5
1.44 ± 0.05	230 ± 30	0.29 ± 0.06	0.361	0.80 ± 0.16	115 ± 5
1.61 ± 0.05	220 ± 30	0.30 ± 0.06	0.373	0.80 ± 0.15	110 ± 5

Supplementary Table 1. **Temperatures and Thomas-Fermi radii to the measurements presented in Fig. 2 (main text).** The temperatures are given in nK as well as units of T_F and T_c. For expressing the temperature in units of T_c we use an interpolated critical temperature curve (see Supp. Fig. 1).

Supplementary Note 2: C-field simulation method

Here we present our simulation method that is used to simulate sound mode dynamics in a condensate of ^{6}Li molecules on the BEC side. The system is described by the Hamiltonian

$$\hat{H}_0 = \int \mathrm{d}\mathbf{r} \Big[\frac{\hbar^2}{2M}\nabla\hat{\psi}^\dagger(\mathbf{r})\cdot\nabla\hat{\psi}(\mathbf{r}) + V(\mathbf{r})\hat{\psi}^\dagger(\mathbf{r})\hat{\psi}(\mathbf{r}) + \frac{g}{2}\hat{\psi}^\dagger(\mathbf{r})\hat{\psi}^\dagger(\mathbf{r})\hat{\psi}(\mathbf{r})\hat{\psi}(\mathbf{r})\Big]. \tag{1}$$

$\hat{\psi}$ and $\hat{\psi}^\dagger$ are the bosonic annihilation and creation operator, respectively. The 3D interaction parameter is given by $g = 4\pi a_\mathrm{dd}\hbar^2/M$, where a_dd is the dimer-dimer scattering length and M the dimer mass. The external potential $V(\mathbf{r})$ represents the cigar-shaped trap $V_\mathrm{trap}(\mathbf{r}) = M(\omega_x^2x^2 + \omega_r^2r^2)/2$. ω_x and ω_r are the axial and radial trapping frequencies, respectively. $r = (y^2+z^2)^{1/2}$ is the radial coordinate.

To perform numerical simulations we discretize space with the lattice of $180\times35\times35$ sites and the discretization length $l = 0.5$ μm, where l is chosen to be smaller than or comparable

to the healing length $\xi > 0.5\,\mu\mathrm{m}$ and the thermal de Broglie wavelength $\lambda_{\mathrm{dB}} \approx 1 - 1.5\,\mu\mathrm{m}$. Since λ_{dB} determines the scale for thermal fluctuations, the associated thermal energy should always be below the cutoff energy introduced by the discretization length. We also note that in the opposite limit $l > \xi$, λ_{dB} the simulation method would be inadequate to capture small-distance excitations, such as vortices. In our c-field representation we replace in Eq. 1 and in the equations of motion the operators $\hat{\psi}$ by complex numbers ψ, see Ref.[4]. We sample the initial states in a grand-canonical ensemble of temperature T and chemical potential μ via a classical Metropolis algorithm. We obtain the time evolution of $\psi(t)$ using the classical equations of motion. As our key observable, we calculate the density $n(\mathbf{r}, t) = |\psi(\mathbf{r}, t)|^2$ and average it over the thermal ensemble. For our simulations we use the trapping frequencies $(\omega_x, \omega_r) = 2\pi \times (70\,\mathrm{Hz}, 780\,\mathrm{Hz})$ that are higher than the experimental trap values. This is because the size of the simulation lattice is needed to be small in order to have a reasonable calculation time. We show below that this larger value of the trapping frequency does not affect our results of the sound velocity, which is determined from the sound propagation near the trap center. We choose the scattering length a_{dd} and the trap central density n_0 according to the experiment. a_{dd} varies in the range $a_{\mathrm{dd}} = 720 - 1650 a_0$, where a_0 is the Bohr radius, and n_0 in the range $n_0 = 5.5 - 11.2\,\mu\mathrm{m}^{-3}$. Together with the trapping frequencies $(\omega_x, \omega_r) = 2\pi \times (70\,\mathrm{Hz}, 780\,\mathrm{Hz})$ these parameters result in a cigar-shaped cloud of $N = 4.0 \times 10^4 - 4.5 \times 10^4$ ^{6}Li molecules. The temperature varies in the range $T = 240 - 280\,\mathrm{nK}$ or $T/T_{\mathrm{c}} = 0.5 - 0.8$.

To excite sound modes we add the perturbation $\mathcal{H}_{ex}(t) = \int \mathrm{d}\mathbf{r}\, V(\mathbf{r}, t) n(\mathbf{r})$, where $n(\mathbf{r})$ is the density at the location $\mathbf{r} = (x, y, z)$. The excitation potential $V(\mathbf{r}, t)$ is given by

$$V(\mathbf{r}, t) = V_0(t) \exp\Big(-\frac{(x - x_0)^2 + (z - z_0)^2}{2\sigma^2}\Big), \tag{2}$$

where $V_0(t)$ is the time-dependent strength and σ is the width. The locations x_0, z_0 are chosen to be the trap center. We excite sound modes following the scheme used in the experiment, where σ and V_0 are chosen such that the changes in the local density due to the excitation potential are consistent with the experiment. We calculate the density profile $\bar{n}_{ex}(x, t)$, which is integrated along the radial direction. For sound propagation we examine $\Delta\bar{n}(x, t) = \big(\bar{n}_{ex}(x, t) - \bar{n}(x)\big)/\bar{n}(0)$, where $\bar{n}(x)$ is the density profile of the unperturbed cloud integrated in the radial direction and $\bar{n}(0)$ is the maximum density. As we show in the main text, the time evolution of $\Delta\bar{n}(x, t)$ displays excitation of second sound, which is identified

by a vanishing sound velocity at R_{TF}. We note that solitonic excitations are not expected as they involve a steep change of the local phase, whereas our excitation protocol modifies the local density. Furthermore the size of solitonic wave features is on the order of the healing length of 0.5 μm whereas the first and second sound features we observe have a width of a few tens of micrometers. We fit $\Delta\bar{n}(x,t)$ with a Gaussian to determine the second sound velocity u_2 at the trap center. We note that within the range of $T/T_{\mathrm{c}} = 0.5-0.7$ the velocity u_2 changes only negligibly with temperature compared to the experimental errorbars.

A. Low versus strong transverse trapping frequency

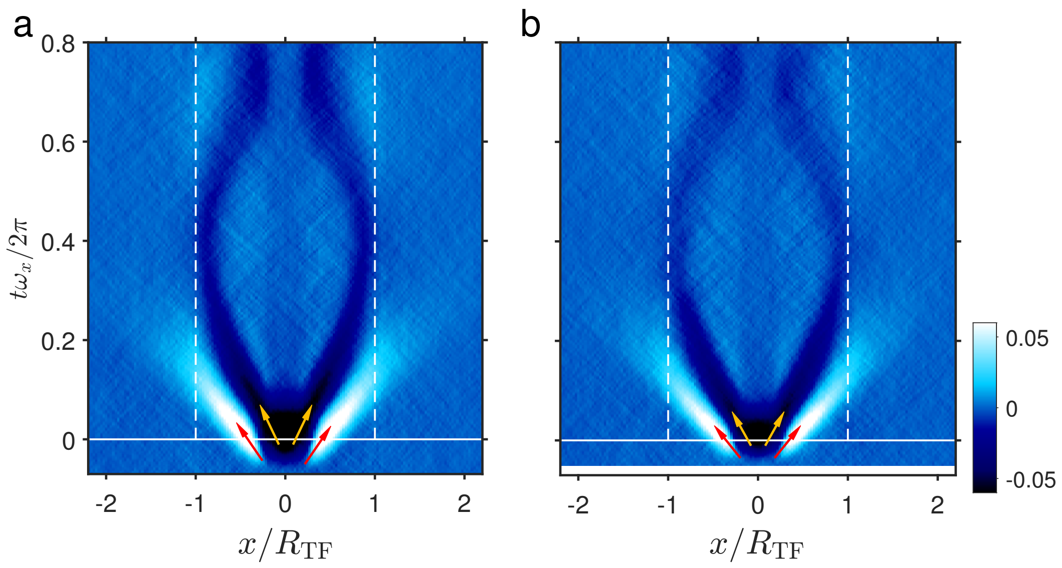

Supplementary Figure 2. **Low versus strong transverse trapping frequency. a,** Time evolution of $\Delta\bar{n}(x,t)$ as a function of axial position x and time t for $\omega_r = 2\pi \times 546\,\mathrm{Hz}$, where sound excitations are created using the excitation frequency $\omega_{\mathrm{ex}}/\omega_r = 0.61$ and a half-cycle modulation. **b,** $\Delta\bar{n}(x,t)$ for $\omega_r = 2\pi \times 780\,\mathrm{Hz}$, where we used the same relative value of $\omega_{\mathrm{ex}}/\omega_r = 0.61$ and the same half-cycle modulation as in the case of **a**. The red and orange arrows indicate the propagation of first and second sound, respectively. The determined values of the second sound velocity u_2 are $(4.74 \pm 0.15)\,\mathrm{mm/s}$ and $(4.5 \pm 0.10)\,\mathrm{mm/s}$ for the systems of low and high trapping frequency, respectively. Consequently, there is at most a weak dependence of the second sound velocity u_2 on the confinement in the transverse direction.

To examine whether a stronger confinement in the transverse direction affects the sim-

ulation result of the sound velocity, we choose a lower transverse trapping frequency of $\omega_r = 2\pi \times 546\,\mathrm{Hz}$ and compare its result with that of $\omega_r = 2\pi \times 780\,\mathrm{Hz}$, while we use the same axial trapping frequency $\omega_x = 2\pi \times 70\,\mathrm{Hz}$ and the same scattering length $a_{\mathrm{dd}} = 840a_0$. The simulated cloud consists of $N \approx 66,000$ and $55,000$ molecules for the systems of low and high trapping frequency, respectively. To excite sound modes we use the excitation frequency $\omega_{\mathrm{ex}}/\omega_r = 0.61$ and a half-cycle modulation, which are the same as in the case of high frequency simulation. In Supp. Fig. 2 we show the time evolution of the density profile for both the systems of low and high trapping frequency. Both simulations show excitation of first and second sound pulses, as indicated by the red and orange arrows in Supp. Fig. 2. The propagation of two sound modes seems similar to the case of high frequency. For a quantitative comparison we determine the second sound velocity u_2, following the procedure described above. We obtain $u_2 = (4.74 \pm 0.15)\,\mathrm{mm/s}$ and $(4.5 \pm 0.10)\,\mathrm{mm/s}$ for the systems of low and high trapping frequency, respectively. This ensures that within the numerical error both systems give a consistent result of the sound velocity.

B. Influence of temperature on the propagation of sound modes

To examine the influence of temperature on sound propagation, we simulate the system at four different temperatures: $T = 120$, 180, 210, and 240 nK, while the trapping frequencies, the scattering length and the central density were kept fixed. This resulted in the number of molecules $N \approx 43000$, 56000, 64000, and 72000 for $T = 120$, 180, 210, and 240 nK, respectively. The healing length at the trap center is around $\xi \approx 0.5\,\mu\mathrm{m}$ and the thermal de Broglie wavelength is in the range $1.0 - 1.5\,\mu\mathrm{m}$, which fulfills the continuum limit assumed in our simulation approach. For all simulations we excite sound modes using one cycle of modulation and the excitation frequency $\omega_{\mathrm{ex}}/\omega_r = 0.61$. In Supp. Fig. 3 we show the time evolution of the density profile $\Delta\bar{n}(x,t)$ for $T = 120$, 180, 210, and 240 nK. At $T = 120 - 210\,\mathrm{nK}$, the time evolution shows multiple sound excitations. We observe two first sound pulses (a bright pulse followed by a dark pulse) that propagate outside the superfluid region. These are created while the excitation scheme is carried out as we discussed in the main text. Following the excitation scheme, also a pulse of second sound is created, which propagates only within the superfluid region and has a vanishing velocity at the Thomas-Fermi radius. In Supp. Fig. 3 a-c (upper row) an additional bright first sound wave

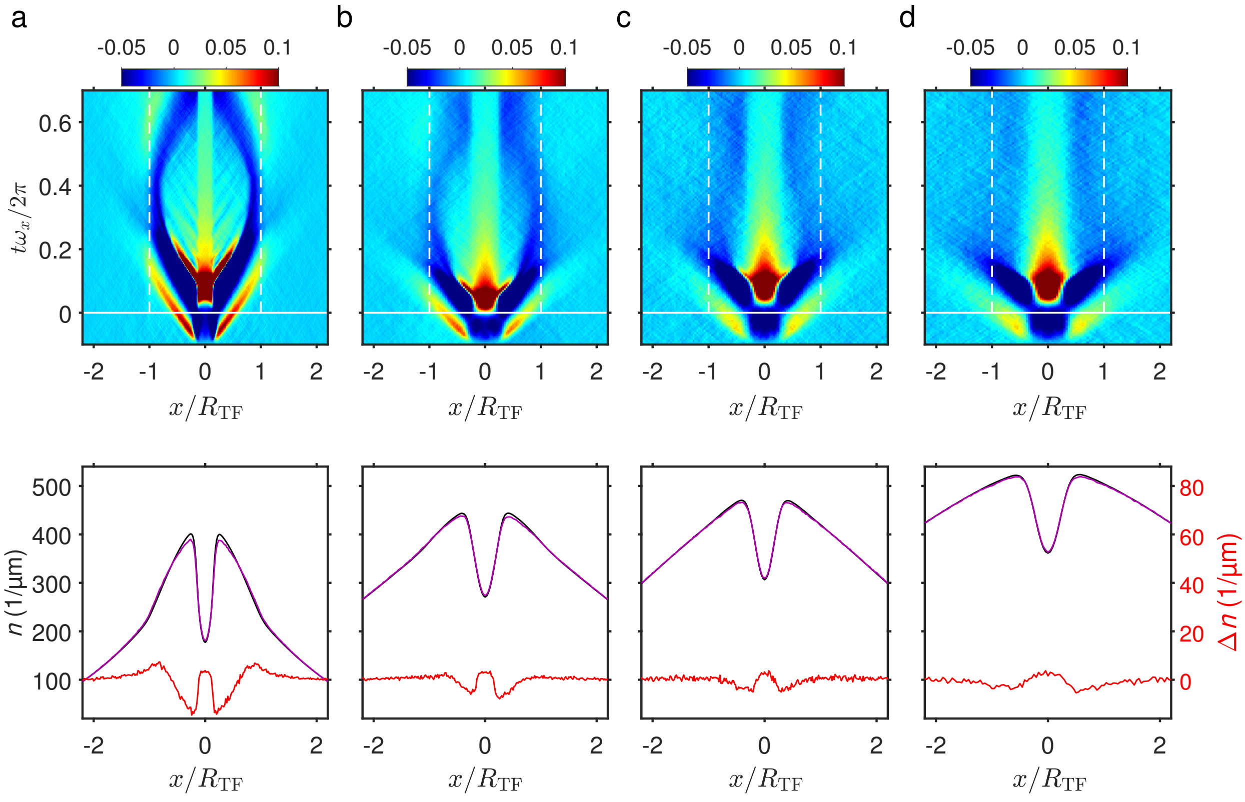

Supplementary Figure 3. **Influence of temperature on the propagation of sound modes. a-d,** Sound excitations created using one cycle of modulation and excitation frequency $\omega_{\mathrm{ex}}/\omega_r = 0.61$ for the temperatures of $T = 120$, 180, 210, and 240 nK, respectively. The bottom row shows the 1D line density profile of the perturbed cloud (purple) at time $t\omega_x/(2\pi) = 0.6$ and the corresponding background density profile (black) as well as their difference Δn (red).

appears which diffuses after a short propagation time. This happens due to the fact that this first sound wave is created after the dark second sound wave but propagates with higher velocity within the superfluid region. When it crosses the second sound wave it fades out in the simulation because mixing of first and second sound leads to diffusion. In addition, second sound signal seems to wash out for increasing temperatures. In fact, at a temperature near the transition temperature second sound becomes a diffusive sound mode as discussed in Ref.[11]. This seems to also increase the diffusion of first sound, leading e.g. to the suppression of the additional bright first sound in Supp. Fig. 3 d.

Although predicted in the simulations the diffusion of first sound is not observed in the experiment (see Fig. 4) as its wave is still clearly visible even beyond the Thomas-Fermi radius. The discrepancy between experiment and theory could be due to the inherent discrete

nature of the simulation method, where the variation in the density and the phase is not smooth on the scale of the discretization length, causing an additional dispersion of the sound wave.

To understand the long time behavior we show in the lower row of Supp. Fig. 3 the 1D density profiles at $t\omega_x/(2\pi) = 0.6$. The purple line corresponds to the cloud where sound was excited. The black line corresponds to the cloud without sound excitation. The red curve is the difference. The peak in the center of the second sound dips is due to the fact that very little or no superfluid phase is present (because the repulsive dipole potential prevents the gas from reaching the critical density in the center, see Supp. Fig. 5) and therefore second sound cannot enter the central region after it gets reflected at the Thomas-Fermi radius. This reflection is strongly visible for the lowest temperature of $T = 120$ nK in Supp. Fig. 3 a (upper row).

Supplementary Note 3: Analytic description of the sound modes

In the following we present an analytic description of first and second sound based on the two-fluid hydrodynamic model for a uniform gas. The total density n of the gas is a sum of the superfluid $n_{\rm s}$ and normal fluid density $n_{\rm n}$. The first and second sound mode squared velocities are given by[5]

$$u_{1/2}^2 = \frac{1}{2}(c_T^2 + c_2^2 + c_3^2) \pm \left[\frac{1}{4}(c_T^2 + c_2^2 + c_3^2)^2 - c_T^2 c_2^2\right]^{1/2}, \tag{3}$$

where $c_T^2 = 1/M(\partial p/\partial n)_T$ and $c_2^2 = n_{\rm s} s^2 T/(n_{\rm n} c_{\rm V})$ representing the isothermal and entropic sound velocities, respectively. p is the pressure, s the entropy per unit mass, T the temperature, and $c_V = T(\partial s/\partial T)_n$ the heat capacity per unit mass. The quantity $c_3^2 \equiv c_S^2 - c_T^2 = (\partial s/\partial n)_T^2 (n^2 T/c_V)$ couples the sound velocities c_2 and c_T, where $c_S^2 = 1/M(\partial p/\partial n)_s$ corresponds to the adiabatic sound velocity. The decoupled sound modes in the limit of vanishing T are

$$u_1^2 = c_T^2 = \frac{1}{M}\left(\frac{\partial p}{\partial n}\right)_T \quad \text{and} \quad u_2^2 = c_2^2 = \frac{n_{\rm s}}{n_{\rm n}} \frac{s^2 T}{c_V}. \tag{4}$$

Here, first and second sound can be described as a pressure and entropy wave, respectively. To determine the second sound velocity u_2, we calculate the entropy and the normal fluid density defined as

$$S = \sum_{\mathbf{k}} \Big(- f_k \log f_k \pm (1 \pm f_k) \log(1 \pm f_k) \Big) \tag{5}$$

and

$$n_{\rm n} = \frac{1}{M} \int \frac{d\mathbf{k}^3}{(2\pi)^3} \frac{\hbar^2 k^2}{3} \Big(- \frac{\partial f_k}{\partial E_k} \Big), \tag{6}$$

respectively[5]. $f_k = 1/\big(\exp(E_k/k_{\rm B}T) \mp 1\big)$ is the thermal occupation number, where E_k is the excitation energy and $\mathbf{k}$ the wavevector. The upper and lower sign correspond to a Bose and Fermi gas, respectively.

A. BEC

We use the Bogoliubov theory, valid in the dilute limit, to analyze the regime $k_{\rm B}T < gn$, where gn is the mean-field energy. The Bogoliubov spectrum is given by $E_k = \sqrt{\epsilon_k(\epsilon_k + 2gn)}$, where $\epsilon_k = \hbar^2 k^2/(2M)$ is the free-particle spectrum. M is the molecular mass. To examine the decoupled modes in Eq. 4 we approximate E_k by the linear spectrum $E_k \approx \hbar c k$, where $c = \sqrt{gn/M}$ is the Bogoliubov sound velocity. We obtain the entropy and the normal fluid density, respectively,

$$S = V \frac{2\pi^2}{45\hbar^3} (k_{\rm B}T)^3 \Big(\frac{M}{gn}\Big)^{3/2} \quad \text{and} \quad n_{\rm n} = \frac{2\pi^2}{45} \frac{(k_{\rm B}T)^4}{\hbar^3} \frac{M^{3/2}}{(gn)^{5/2}}. \tag{7}$$

The entropy per unit mass is $s = S/(NM) = gn_{\rm n}/(MT)$ and the heat capacity per unit mass is $c_V = 3s$.

Within upper description we can deduce following sound speeds

$$u_1 = \sqrt{\frac{gn}{M}} \quad \text{and} \quad u_2 = \sqrt{\frac{1}{3}\frac{gn}{M}}. \tag{8}$$

Here, u_2 is $u_1/\sqrt{3}$. This result is only valid at zero temperature, see Supp. Fig. 4a, where we show the full numerical solutions of Eq. 3 using the Bogoliubov description.

For $k_{\rm B}T > gn$ instead we make use of a thermal gas description to determine s, c_V, and $n_{\rm n}$, which are given by $s = 2.568 k_{\rm B} n_{\rm n}/(2Mn)$, $c_V = 3s/2$, and $n_{\rm n} = n(T/T_{\rm c})^{3/2}$, respectively[5]. In this regime, solving Eq. 3 the sound velocities read,

$$u_1 = \sqrt{\frac{gn}{M} + \frac{0.856 k_{\rm B}T}{M}} \quad \text{and} \quad u_2 = \sqrt{\frac{n_{\rm s}}{n}\frac{gn}{M}}. \tag{9}$$

u_2 is proportional to $\sqrt{n_\mathrm{s}/n}$ and can be approximated by $u_2 = \sqrt{\left(1-(T/T_\mathrm{c})^{3/2}\right)gn/M}$ (see Supp. Fig. 4a).

Sound amplitudes

Besides the sound velocity, our analytic description can be used to determine the amplitudes of the propagating sound modes, described as[6]

$$\delta n(x,t) = W_1 \delta\tilde{n}(x \pm u_1 t) + W_2 \delta\tilde{n}(x \pm u_2 t). \tag{10}$$

where $\delta\tilde{n}(x,t)$ is the density variation created by the excitation potential. $\delta\tilde{n}(x \pm u_{1/2}t)$ represent wave packets of first and second sound with weights $W_{1/2}$. The relative weight is given by[6]

$$\frac{W_2}{W_1} = \frac{c_2^2 - u_2^2}{u_1^2 - c_2^2}\frac{u_1^2}{u_2^2} \tag{11}$$

We determine W_2/W_1 by numerically solving Eq. 3 for the regimes $k_\mathrm{B}T < gn$ and $k_\mathrm{B}T > gn$ using the Bogoliubov and thermal gas description, respectively.

We show these results in Supp. Fig. 4b. The Bogoliubov description of the weight works only for $k_BT \ll gn$. We note that at higher temperatures terms beyond Bogoliubov are needed to account for the thermal damping of the modes. The Bogoliubov description thus leads to an overestimation of the weight at high temperatures. For temperatures above the mean-field energy the weight is described by the thermal gas description, which we use to estimate the relative weight of the two modes in the main text. Please note that the thermal description gives unphysical solutions for $k_BT/gn \to 1$. In the experiment presented in fig. 3 of the main text $k_BT/gn \approx 0.8$ in the central region and therefore the Bogoliubov description should be valid.

B. BCS

A condensate of an interacting Fermi gas is described by the BCS spectrum $E_k = \sqrt{\xi_k^2 + \Delta^2}$, with $\xi_k = \hbar^2 k^2/(2m) - \mu$, where μ is the chemical potential and $\Delta(T)$ the gap. At low $k_\mathrm{B}T \ll \Delta$, we use $\mu \approx E_\mathrm{F}$ and expand ξ_k near the Fermi surface, i.e.

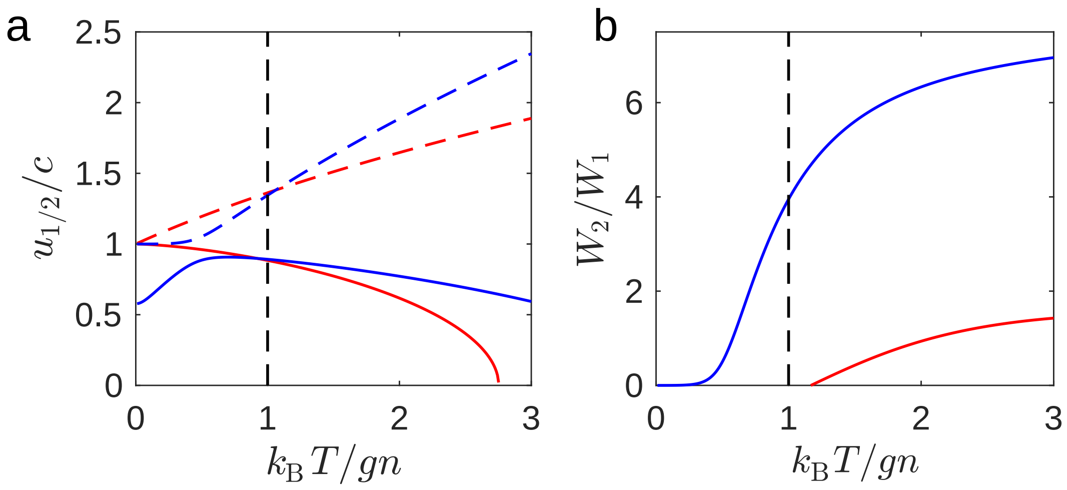

Supplementary Figure 4. **Sound velocities and amplitudes.** **a,** Sound velocities $u_{1/2}$ are determined from Eq. 3 and are shown as a function of $k_\mathrm{B}T/gn$ using the Bogoliubov (blue lines) and thermal gas description (red lines). Here, c is the Bogoliubov sound speed introduced in the text. **b,** shows the relative weight W_2/W_1 for $k_\mathrm{B}T < gn$ (blue line) and $k_\mathrm{B}T > gn$ (red line). In the experiment in fig. 3 of the main text $k_BT/gn \approx 0.8$ in the central region.

$\xi_k = \hbar^2k^2/(2m) - E_\mathrm{F} \approx \hbar v_\mathrm{F}|k - k_\mathrm{F}|$ (see Ref.[7]). The entropy in Eq. 5 results in

$$S = \frac{3N_\mathrm{tot}}{E_\mathrm{F}} \int_0^\infty d\xi_k \frac{E_k}{k_\mathrm{B}T} \exp\Big(-\frac{E_k}{k_\mathrm{B}T}\Big) = 3N_\mathrm{tot}\frac{\Delta_0}{E_\mathrm{F}}\sqrt{\frac{\pi\Delta_0}{2k_\mathrm{B}T}} \exp\Big(-\frac{\Delta_0}{k_\mathrm{B}T}\Big), \tag{12}$$

with

$$\Delta_0 = (2/e)^{7/3}E_\mathrm{F} \exp\big(\pi/(2k_\mathrm{F}a)\big) \tag{13}$$

which is the gap at zero temperature[8]. With Eq. 12 we determine $s = S/(mN_\mathrm{tot})$ and c_V. The normal fluid density in Eq. 6 gives

$$\frac{n_\mathrm{n}}{n_\mathrm{tot}} = 2\int_0^\infty d\xi_k \left(-\frac{\partial f_k}{\partial E_k}\right) = \sqrt{\frac{2\pi\Delta_0}{k_\mathrm{B}T}} \exp\Big(-\frac{\Delta_0}{k_\mathrm{B}T}\Big). \tag{14}$$

Using s, c_V, and n_n in Eq. 4 we obtain the second sound velocity

$$u_2 = \frac{\sqrt{3}}{2}\frac{k_\mathrm{B}T}{E_\mathrm{F}}v_\mathrm{F}, \tag{15}$$

which is valid for $T < T_\mathrm{c}$. The BCS critical temperature is given by $k_\mathrm{B}T_\mathrm{c} = (\gamma/\pi)\Delta_0 = 0.567\Delta_0$, which depends on the interaction parameter $(k_\mathrm{F}a)^{-1}$. We show in the main text the result u_2 at various interactions on the BCS side (see Fig. 2). u_2 vanishes at zero

temperature contrary to the BEC superfluids. We note that this result is consistent with Ref.[9].

Supplementary Note 4: BEC mean-field model

To estimate the density distribution of a partially Bose condensed cloud in the BEC regime we carry out a self-consistent calculation where the condensate phase is treated within the Thomas-Fermi approximation and for the normal phase we use a standard thermodynamical approach. Specifically, we solve the following set of coupled equations[10]

$$n_{\mathrm{s}}(\mathbf{r}) = \frac{\mu_{\mathrm{s}} - V_{\mathrm{ext}}(\mathbf{r}) - 2gn_{\mathrm{n}}(\mathbf{r})}{g}\Theta\left(\mu_{\mathrm{s}} - V_{\mathrm{ext}}(\mathbf{r}) - 2gn_{\mathrm{n}}(\mathbf{r})\right) \tag{16}$$

$$n_{\mathrm{n}}(\mathbf{r}) = \frac{1}{\lambda_{\mathrm{dB}}^3}\mathrm{Li}_{3/2}\left(\exp\left\{\frac{\mu_{\mathrm{n}} - V_{\mathrm{ext}}(\mathbf{r}) - 2gn_{\mathrm{s}}(\mathbf{r}) - 2gn_{\mathrm{n}}(\mathbf{r})}{k_B T}\right\}\right). \tag{17}$$

Here, λ_{dB} is the thermal de Broglie wavelength, $g = 4\pi\hbar^2 a_{\mathrm{dd}}/M$ is the coupling constant, T is the temperature and $V_{\mathrm{ext}}(\mathbf{r})$ is the external potential consisting of the harmonic trapping potential and the repulsive potential of the excitation beam, μ_{s} and μ_{n} are the chemical potentials of the superfluid and the normal fluid part, respectively. For the calculation we set $\mu_{\mathrm{n}} = \min[V_{\mathrm{ext}}(\mathbf{r}) + 2gn_{\mathrm{s}}(\mathbf{r}) + 2gn_{\mathrm{n}}(\mathbf{r})]$ which ensures that the normal gas reaches the critical density $n_{\mathrm{n,crit}} = \mathrm{Li}_{3/2}(1)/\lambda_{\mathrm{dB}}^3$ at the Thomas-Fermi radius. This way, the number of normal fluid atoms is fixed. μ_{s} is chosen such that the total atom number matches the experimental value.

Equation 16 represents the Thomas-Fermi approximation where we take into account the repulsive mean-field potential of the normal fluid part. Equation 17 is the density distribution of a thermal bosonic cloud, again including the additional mean-field potential produced by the atoms. By self-consistently solving the coupled equations we obtain the density distributions of the superfluid and the normal fluid gas as shown in Supp. Fig. 5. The repulsive excitation beam pushes the atoms away from the trap center which creates a density profile with two peaks of the same height. Interestingly in our self-consistent calculations we find that the peak density with and without the excitation beam is almost the same. This holds both for the line density and the 3D density. This allows for extracing the peak density in our experiments, when the excitation laser is present, from reference absorption images of an unperturbed cloud when no excitation laser is present. To do this,

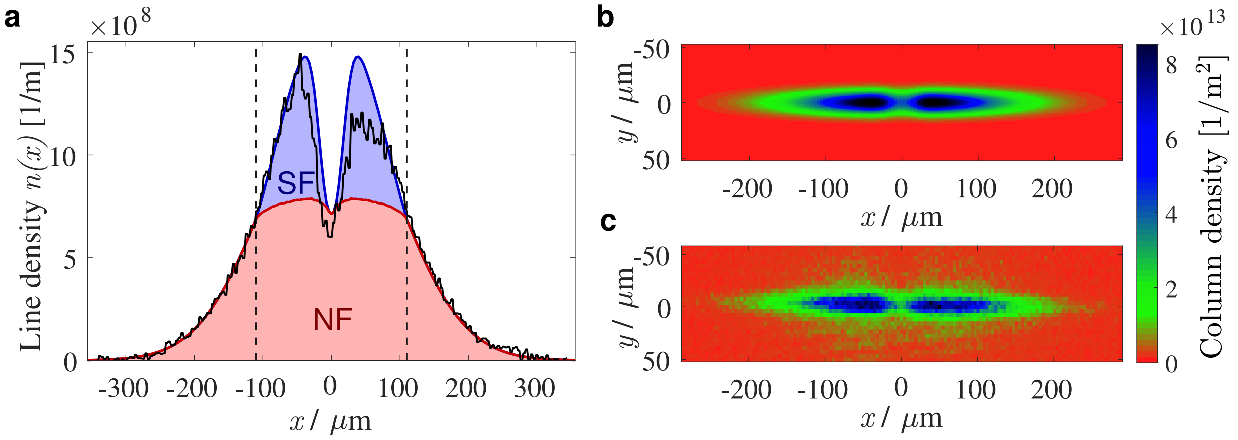

Supplementary Figure 5. **Calculated and measured atom densities at $(k_\mathrm{F}a)^{-1} = 1.61$ and a temperature of $T = 220\,\mathrm{nK}$. The repulsive excitation laser beam in the trap center locally reduces the atom density. The shown density distributions correspond to the state just before modulation starts.** **a**, The blue and red line show the line densities of the superfluid and the normal phase obtained from a self-consistent calculation, the measured total line density is shown is black. The vertical dotted lines indicate the Thomas-Fermi radius at $x = \pm 110\,\mu\mathrm{m}$. **b**, Calculated absorption image of the atom cloud. **c**, Measured absorption image of the atom cloud.

we apply the inverse Abel transformation to the reference absorption images to reconstruct the 3D density profile. Note that this transformation is in generally valid only for rotationally symmetric clouds, which is the case when no exciation beam is present. We have verified that we obtain the same density using our self-consistent calculations where we input the temperature, the total number of atoms, the trapping frequencies and the scattering length. This is possible since the interaction parameter of $(k_\mathrm{F}a)^{-1} = 1.61$ is still close to the BEC regime. Supplementary Figure 5 a-c shows there is good agreement for the calculated and measured density distributions, which validates this approach.

SUPPLEMENTARY REFERENCES

[1] Paintner, T. et al. Pair fraction in a finite-temperature Fermi gas on the BEC side of the BCS-BEC crossover. *Phys. Rev. A* **99**, 053617 (2019).

[2] Pini, M., Pieri, P., Jäger, M., Hecker Denschlag J., and Calvanese Strinati, G. Pair correlations in the normal phase of an attractive Fermi gas. *New J. Phys.* **22** 083008 (2019).

[3] Ku, M. J. H., Sommer, A. T., Cheuk, L. W. and Zwierlein, M. W. Revealing the Superfluid Lambda Transition in the Universal Thermodynamics of a Unitary Fermi Gas. *Science* **335**, 563 (2012).

[4] Singh, V. P. et al. Probing superfluidity of Bose-Einstein condensates via laser stirring. *Phys. Rev. A* **93**, 023634 (2016).

[5] Pethick, C. J. and Smith, H. *Bose-Einstein Condensation in Dilute Gases.* (Cambridge University Press, Cambridge, 2008)

[6] Arahata, E. and Nikuni, T. Propagation of second sound in a superfluid Fermi gas in the unitary limit. *Phys. Rev. A* **80**, 043613 (2009).

[7] Lifshitz, E.M. and Pitaevskii, L.P. *Statistical Physics, Part 2, Theory of the Condensed State.* (Butterworth-Heinemann, Oxford, 1980).

[8] Gor'kov, L.P. and Melik-Barkhudarov, T.K. Contribution to the Theory of Superfluidity in an Imperfect Fermi Gas. *Sov. Phys. JETP* **13**, 1018 (1961).

[9] Heiselberg, H. Sound modes at the BCS-BEC crossover. *Phys. Rev. A* **73**, 013607 (2006).

[10] Pitaevskii, L. and Stringari, S. *Bose-Einstein Condensation.* (Oxford University Press, Oxford, 2003).

[11] Singh, V. P. and Mathey, L. Sound propagation in a two-dimensional Bose gas across the superfluid transition. *Phys. Rev. Research* **2** 023336 (2020).
